# Early Diagnostic Classification of Alzheimer’s Disease Using Structural and Resting-State Functional MRI

**DOI:** 10.3390/neurolint18100191

**Published:** 2026-10-13

**Authors:** Junhu Liu, Bing Zhu, Miao Yu, Shichao Du

**Affiliations:** Jilin Provincial Key Laboratory for Numerical Simulation, Jilin Normal University, Siping 136000, China

**Keywords:** Alzheimer’s disease, sMRI, resting-state fMRI, Transformer, graph theory

## Abstract

**Background**: Alzheimer’s disease (AD) is an irreversible neurodegenerative disorder where early diagnosis at the mild cognitive impairment (MCI) stage is critical for timely intervention. While structural MRI (sMRI) and resting-state functional MRI (rs-fMRI) provide complementary pathological information, clinical translation is limited by high feature heterogeneity and the heavy computational burden of 3D deep learning networks. This study aims to propose a lightweight, hybrid multimodal fusion model to overcome these limitations and improve diagnostic efficiency. **Methods**: A lightweight MobileViT-XS network was utilized to extract global and local pathological features from gray-matter sMRI images to reduce computational costs. Concurrently, graph theory was applied to rs-fMRI data to construct brain functional networks and calculate global topological properties. Following pretraining, feature-level fusion was performed to integrate sMRI features, rs-fMRI properties, demographic information, and two clinical neuropsychological examinations. **Results**: The validation results show that the proposed hybrid model achieved high diagnostic performance in the three-class classification task, yielding an accuracy of 0.928 ± 0.010, sensitivity of 0.901 ± 0.014, precision of 0.933 ± 0.014, and an F1 score of 0.911 ± 0.012. On the held-out testing subset of the independently collected dataset after dataset-specific fine-tuning, the proposed model achieved an accuracy of 0.857, with a sensitivity of 0.867, a precision of 0.867, and an F1 score of 0.859. **Conclusions**: The proposed framework successfully minimizes computational overhead while maintaining high classification accuracy. These findings suggest that the hybrid multimodal fusion model provides a promising computer-aided approach for three-class classification of AD, MCI, and NC.

## 1. Introduction

Alzheimer’s disease (AD) is an irreversible neurodegenerative disorder. It is clinically characterized by memory decline and cognitive impairment. In severe cases, patients may lose the ability to live independently. The prevalence of AD increases with age. It has been estimated that more than 55 million people worldwide are living with dementia, and this number continues to rise [1]. Mild cognitive impairment (MCI) is a critical transitional stage between normal controls (NC) and AD. Although patients with MCI exhibit mild cognitive decline, their activities of daily living are generally preserved. Therefore, this stage is considered an optimal window for early intervention and treatment of AD [2]. However, the pathogenesis of AD remains incompletely understood, and the disease is often difficult to reverse once it progresses to the dementia stage. Therefore, accurate identification and timely intervention at the early stage of AD are of great significance for delaying disease progression and improving patients’ quality of life.

In the field of non-invasive imaging-based diagnosis, the combined use of structural magnetic resonance imaging (sMRI) and resting-state functional magnetic resonance imaging (rs-fMRI) provides a new approach for the early diagnosis of AD [3]. sMRI provides high-resolution structural images of the brain. It can reflect AD-related anatomical alterations and help assess the degree of brain atrophy. It can also provide useful information for identifying the stage of disease progression [4,5]. Deep learning algorithms based on sMRI images have been widely used in AD detection models [6]. rs-fMRI reveals abnormal changes in functional connectivity and neural activity patterns by detecting spontaneous fluctuations in blood oxygen level-dependent (BOLD) signals [7]. Graph theory-based functional brain network analysis has become an important direction in rs-fMRI studies [8]. In this approach, the whole brain is parcellated using brain atlases such as the Anatomical Automatic Labeling (AAL) template. Brain regions are defined as network nodes, and functional connections between regions are defined as network edges. Topological properties are then calculated, including global efficiency, small-worldness, characteristic path length, clustering coefficient, nodal efficiency, and betweenness centrality. These measures can quantitatively characterize the information transmission efficiency and organizational features of brain networks [9,10]. Studies have shown that patients with AD exhibit abnormal topological organization of functional brain networks even at the early stage of the disease. Therefore, graph theory-derived brain network features have become important imaging markers for early computer-aided diagnosis of AD. A large body of evidence suggests that sMRI can characterize brain structural degeneration in AD, whereas rs-fMRI can reveal early functional network reorganization [11,12]. Because these two modalities provide complementary structural and functional information, their integration may improve the early diagnosis of AD. However, effectively fusing structural and functional data remains a key challenge in intelligent AD diagnosis due to substantial differences in data dimensionality, representation, and statistical properties.

In clinical practice, the diagnosis of AD is essentially a multimodal decision-making process. Clinicians usually need to integrate medical history, clinical neuropsychological examinations, and multiple imaging findings to obtain a comprehensive understanding of disease progression. Therefore, a single data modality is often insufficient to fully characterize the disease status [13,14]. Previous studies have shown that multimodal data fusion can significantly improve AD classification performance and disease prediction ability [15]. For example, integrating MRI imaging features with demographic information, clinical scales, genetic information, and other data can capture disease-related information from multiple dimensions, including brain structure, brain function, and clinical manifestations [16,17]. This can further improve the diagnostic accuracy and generalization ability of the model.

With the development of artificial intelligence, deep learning has gradually become an important research direction for computer-aided diagnosis of AD [18]. Unlike traditional machine learning methods that rely on manually designed features, deep learning can automatically learn high-level feature representations from data through end-to-end training. It has achieved remarkable success in image classification, object detection, and medical image analysis [19]. Convolutional neural networks (CNNs) can effectively extract local spatial features from brain images. They can also provide better flexibility and generalization by fine-tuning the network architecture for medical datasets. Therefore, CNNs have been widely used in AD classification studies [19,20]. However, the inherent local receptive field of conventional CNNs limits their ability to capture whole-brain anatomical associations and global functional network dependencies. In recent years, Transformer models have shown excellent performance in natural language processing and computer vision due to their self-attention mechanism. They can effectively capture global contextual information. This provides a new solution for modeling complex brain network patterns [21]. The attention mechanism can emphasize regions of interest in the network by autonomously learning a set of parameters [22]. Multi-head self-attention further extends the self-attention mechanism. It uses multiple attention heads in parallel to capture different relationships and features. In particular, the introduction of the Vision Transformer (ViT) has shown great potential for medical image classification tasks [23]. ViT applies a Transformer-like architecture to image patches. When trained on large-scale image datasets, it can outperform common CNN architectures [24,25]. Researchers have gradually recognized its great potential in computer vision tasks such as image classification. To capture global associations, many models have introduced three-dimensional whole-brain self-attention architectures [24]. For example, Khosravi et al. designed a hybrid cascaded attention model (CAM-CNN) [26]. Sarraf et al. proposed an optimized ViT architecture for AD, namely OViTAD. These models performed end-to-end prediction directly on three-dimensional whole-brain images. They effectively improved classification accuracy [27]. However, methods that directly process high-dimensional whole-brain data usually require high computational and memory costs. To reduce computational complexity, some studies have turned to slice-based or patch-based modeling. Hu et al. designed Conv-Swinformer and VGG-TSwinformer [28,29]. These models use CNNs and Swin Transformer to extract features from 2D slices and longitudinal time series. To capture more subtle local lesions, Zhang et al. proposed a multiscale residual analysis framework based on local 3D image patches [30]. Tang et al. developed the CsAGP dual-Transformer model by combining cross-modal self-attention with graph pooling. This model strengthened deep cross-modal interaction through local 3D image patches and graph pooling [31]. Nevertheless, slice-based methods may ignore the spatial continuity and anatomical relationships between adjacent voxels. Local patch partitioning may also artificially separate brain subregions. In addition, these methods highly depend on accurate registration of heterogeneous images. As a solution, Hoang et al. used ViT to model local features of sMRI images in parallel. Based on pretrained weights, they classified and predicted sMRI data from patients with AD and MCI, achieving an accuracy of 83.27 [32]. Furthermore, ViT can also effectively address multimodal classification tasks. It has been increasingly applied by researchers to different datasets [33,34].

However, Transformer models usually have a large number of parameters and high computational costs. They also require substantial hardware resources, which limits their practical deployment and wider application in clinical settings [35]. To balance model performance and computational efficiency, lightweight networks have gradually become a research focus. MobileViT is a Transformer-based visual attention model. Inspired by the design of lightweight CNNs, it is a lightweight and general-purpose ViT suitable for mobile devices [36]. As a lightweight visual model that combines the strengths of convolutional neural networks and Transformers, MobileViT preserves the ability to extract local texture features while enabling global feature modeling. In MobileViT, lightweight CNN modules can effectively capture local image features and texture information. They reduce the network size and improve latency while maintaining high classification performance. They are also widely applicable and easy to train. These modules provide more expressive feature representations for the subsequent Transformer modules. The ViT modules in MobileViT are responsible for global feature modeling and contextual relationship construction. Unlike standard ViT models, which lack strong spatial inductive bias, MobileViT encodes global information while preserving the patch and spatial pixel orders of the input tensor. This design helps the model better capture local structural patterns and spatial relationships. Therefore, MobileViT combines local convolution with global information encoding. This design significantly reduces the number of model parameters and computational complexity while maintaining good performance. At present, MobileViT has also been applied in several medical image classification studies and has achieved promising results [37,38].

Based on the above analysis, this study develops an intelligent diagnostic method based on multimodal data fusion for early AD identification. To better extract local brain features in patients with AD, gray-matter images derived from preprocessed sMRI scans were used as one classification modality [39,40]. For rs-fMRI images, functional brain network matrices were constructed using graph theory, and global brain network properties were calculated [41]. These global network properties were combined with dimensionally compatible demographic information and clinical neuropsychological examinations to build a multidimensional feature system. Subsequently, MobileViT was used to extract sMRI image features, and a multilayer perceptron (MLP) was used to learn non-imaging feature representations. Feature-level fusion was then performed to achieve collaborative modeling of multimodal information. Then, the fused features were used to construct an early diagnostic classification model for AD, MCI, and NC. Finally, the proposed framework was evaluated using a public ADNI dataset and an independently collected dataset, with the latter used for held-out testing after dataset-specific fine-tuning. The results provide an assessment of classification performance under the evaluated data-partitioning settings.

## 2. Materials and Methods

### 2.1. Participants

**Figure 1:**
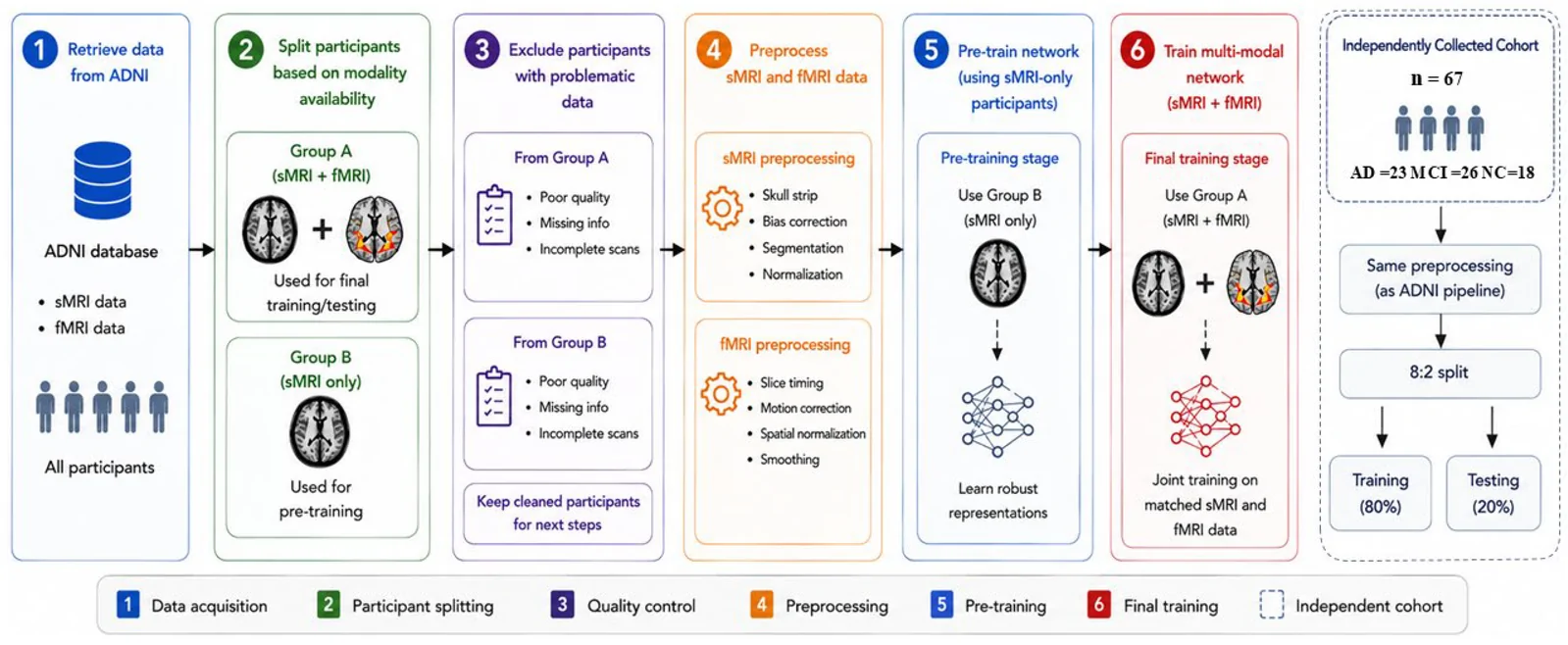
Flowchart of data selection and cohort partitioning for the ADNI and independently collected datasets. Participants were categorized into two independent and non-overlapping cohorts based on modality availability and quality control criteria. Group A serves as the multimodal final training and testing set consisting of participants with complete time-matched sMRI, rs-fMRI, demographic, and clinical examination scores. Group B serves as the single-modality pre-training set comprising independent participants with sMRI data whose remaining modalities were incomplete, ensuring strict participant-level separation between pre-training and multimodal evaluation. An independently collected dataset of 67 participants was additionally included for held-out testing after dataset-specific fine-tuning.

To comprehensively evaluate the classification performance and generalization ability of the proposed lightweight multimodal fusion model, data were obtained from the ADNI 2 and ADNI GO cohorts within the ADNI database. As shown in the data flowchart in Figure 1 and Table 1, participants were categorized into two independent and non-overlapping cohorts based on modality availability and quality control criteria. Specifically, the multimodal final training and testing set, designated as Group A, includes participants with complete and time-matched structural magnetic resonance imaging, resting-state functional magnetic resonance imaging, demographic information such as age, sex, and weight, as well as clinical examination scores including Mini-Mental State Examination (MMSE) and Clinical Dementia Rating (CDR). Following quality control exclusions for poor image quality, missing clinical neuropsychological examination scores, or severe motion artifacts, Group A retained 294 unique participants, comprising 88 patients with AD, 115 patients with MCI, and 91 NC. Meanwhile, the single-modality pre-training set, designated as Group B, includes independent participants with structural magnetic resonance imaging data whose remaining modalities were incomplete or unassigned. Following quality control filtering, Group B retained 1383 structural magnetic resonance imaging scans from 776 unique participants, including 252 scans from 147 AD participants, 480 scans from 325 MCI participants, and 651 scans from 304 NC. Strict participant-level separation was enforced, ensuring that no participant in Group B was included in Group A and thereby guaranteeing zero overlap between the pre-training stage and the multimodal evaluation stage.

In addition, an independently collected dataset comprising 67 participants, including 23 AD, 26 MCI, and 18 NC participants, was obtained from the First Hospital of Jilin University. The demographic and clinical characteristics of this dataset are summarized in Table 1. Complete and matched sMRI, rs-fMRI, demographic, and neuropsychological data were available for all participants. The same preprocessing and feature extraction procedures as those used for the ADNI dataset were applied. The participants were randomly divided at the participant level into a model-fitting subset and a held-out testing subset at an 8:2 ratio, while maintaining a relatively consistent class distribution. The pretrained MobileViT-XS sMRI branch obtained from the ADNI sMRI-only pre-training stage was used to initialize the model, and the model-fitting subset was subsequently used for dataset-specific fine-tuning. The held-out testing subset was not used for model optimization or parameter selection and was reserved exclusively for final evaluation.

### 2.2. sMRI Data Acquisition

sMRI images of the participants were acquired using 3.0T Philips and Siemens MRI scanners across multiple ADNI datasets. To account for potential hardware heterogeneity, the distribution of scanner manufacturers and magnetic field strengths was carefully evaluated across the diagnostic groups. A three-dimensional magnetization-prepared rapid gradient-echo (3D MPRAGE) sequence was used for image acquisition. The acquisition parameters were as follows: repetition time (TR) = 2300 ms, echo time (TE) = 3.0 ms, flip angle (FA) = 9.0°, field-of-view (FOV) = 240 × 256 × 176 mm^3^, acquisition matrix = 256 × 256, slice thickness = 1.2 mm, and number of slices = 160. For the independently collected dataset, sMRI data were acquired using a 3.0 T Philips MRI scanner with a 3D MPRAGE sequence. The acquisition parameters were as follows: TR = 7 ms, TE = 3.3 ms, FA = 7°, FOV = 256 × 256 × 192 mm^3^, acquisition matrix = 256 × 256, slice thickness = 1.0 mm, and 192 slices.

### 2.3. rs-fMRI Data Acquisition

When screening rs-fMRI data from the ADNI dataset, the scanning parameters varied across different acquisition batches. Therefore, the scanning parameters of the three participant groups were categorized and summarized according to the TR and TE settings. The results are shown in Table 2. The matrix size was 64 × 64 for all scans. For the independently collected dataset, rs-fMRI data from the independently collected dataset were acquired using the same 3.0 T Philips MRI scanner. The acquisition parameters were TR = 2000 ms, TE = 30 ms, FA = 90°, FOV = 224 × 224 × 138 mm^3^, matrix size = 64 × 64, slice thickness = 3.5 mm, and 33 slices.

### 2.4. sMRI Preprocessing

The preprocessing of sMRI was performed using the Computational Anatomy Toolbox (CAT12, http://www.neuro.uni-jena.de/cat/ (accessed on 15 November 2025)) and the Statistical Parametric Mapping (SPM12, http://www.fil.ion.ucl.ac.uk/spm/ (accessed on 15 November 2025)). The sMRI images of the three participant groups were segmented using the International Consortium for Brain Mapping (ICBM) Space Template—European Brains. After preprocessing, the sMRI volume was 121 × 145 × 121 voxels and was resampled to 121 × 256 × 256 voxels using trilinear interpolation before network input. Finally, the three-dimensional gray-matter images after image registration and gray-matter volume modulation were selected as the input features of the network model. The independently collected dataset underwent the same preprocessing pipeline as the ADNI dataset, including spatial normalization, resampling, and subsequent feature extraction procedures, to ensure consistency between the two datasets.

### 2.5. rs-fMRI Preprocessing and Functional Brain Network Features

To account for variations in scanning parameters across participants, data with different parameter settings were preprocessed in separate batches using SPM12. Following the removal of the first 10 time points, slice timing correction and realignment were applied. The images were normalized to MNI space with a voxel size of 3 × 3 × 3 mm^3^ and spatially smoothed using an 8 mm FWHM Gaussian kernel. Temporal preprocessing involved linear detrending and bandpass filtering from 0.01 to 0.10 Hz. To mitigate non-neural artifacts, signals from white matter (WM), cerebrospinal fluid (CSF), and the global brain were regressed out. Head motion effects were modeled using the Friston 24-parameter model. Framewise displacement (FD) was used to assess head motion, and motion scrubbing was performed using a threshold of 0.5 mm by removing one preceding and two subsequent time points for each suprathreshold point.

The functional brain network was constructed using the AAL90 template as the region of interest (ROI). The AAL90 template is widely adopted in AD functional brain network studies because it provides a stable and anatomically interpretable parcellation of the cerebral cortex and major subcortical structures [42]. This parcellation provides sufficient brain coverage while controlling feature dimensionality and computational complexity, thereby facilitating research comparability and interpretability. Following preprocessing, mean time series were extracted from the 90 regions defined by the AAL90 atlas, and pairwise Pearson correlation coefficients were calculated using the GRETNA toolbox to construct individual 90 × 90 functional connectivity matrices [43].

Fisher’s r-to-z transformation was then applied to the correlation coefficients to improve normality. During this process, only positive correlations were retained, whereas negative correlations were excluded. Binary functional networks were generated using network sparsity thresholds ranging from 0.10 to 0.50 with an increment of 0.01. For each sparsity level, 100 random networks were generated as reference networks for normalized graph measures. The mean area under the curve across the sparsity range was calculated for subsequent statistical analysis. Based on graph theory, 10 global brain network properties were calculated from the obtained matrices. These global properties included the normalized clustering coefficient (γ), normalized characteristic path length (λ), small-worldness (σ), clustering coefficient (C_p_), characteristic path length (L_p_), local efficiency (E_loc_), global efficiency (E_g_), assortativity, synchronization, and hierarchy. The global properties are described in Table 3. The global brain network properties were used as feature data input to the network model.

### 2.6. Multimodal Fusion Classification Network Architecture

**Figure 2:**
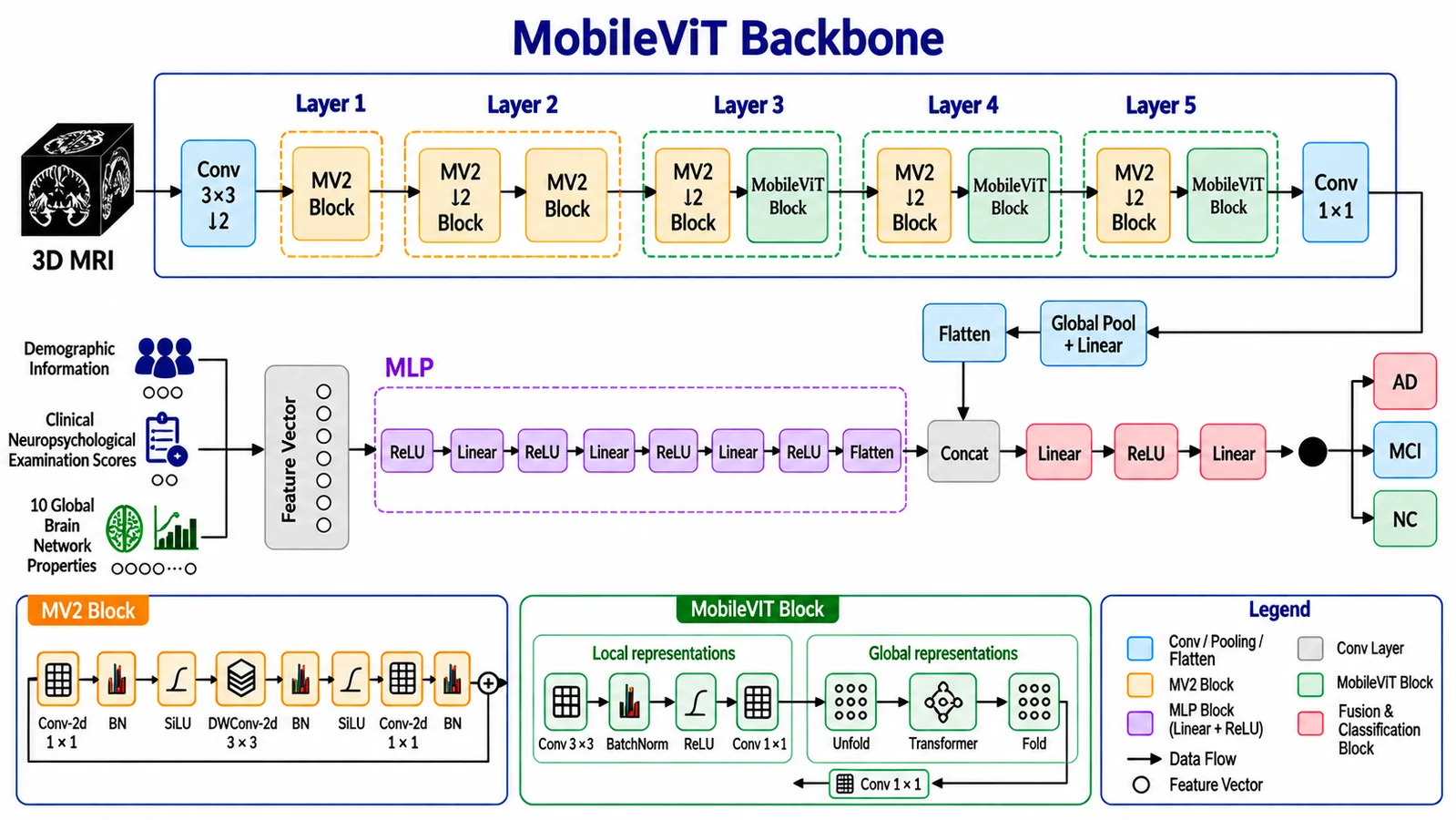
Overall architecture of the proposed MobileViT-based multimodal classification framework. The 3D sMRI volume was represented as 121 axial slices and arranged as input channels for the 2D MobileViT backbone. The backbone consisted of an initial convolutional stem, MV2 blocks, and MobileViT blocks, followed by global pooling. Demographic and clinical features were processed using an MLP and subsequently fused with the image representation for three-class classification of AD, MCI, and NC. The notation ↓2 indicates downsampling by a factor of two.

To achieve effective diagnostic classification of AD, a feature-level multimodal fusion network was constructed, as shown in Figure 2. The 3D sMRI gray-matter images were processed using the MobileViT backbone, while the non-imaging numerical variables were processed using a multilayer perceptron. The non-imaging input was a 15-dimensional vector for each participant, comprising 3 demographic variables, 2 clinical examination scores, and 10 global brain network properties derived from rs-fMRI. The MLP consisted of four sequential linear layers containing 32, 64, 128, and 64 units, respectively, with a ReLU activation function following each linear layer. In this study, the preprocessed sMRI volume of 121 × 256 × 256 voxels was represented as a tensor of shape (B, 121, 256, 256), treating the 121 axial slices as input channels. The initial convolution used a 3 × 3 kernel with stride 2 and padding 1, producing a feature map of (B, 16, 128, 128). The subsequent stages produced feature maps of (B, 32, 128, 128), (B, 48, 64, 64), (B, 64, 32, 32), (B, 80, 16, 16), and (B, 96, 8, 8). The MobileViT blocks used non-overlapping 2 × 2 patches, with Transformer embedding dimensions of 96, 120, and 144 in Layers 3 through 5, respectively.

Based on the characteristics of the gray-matter data extracted from the sMRI images of the three participant groups, MobileViT was selected as the feature extraction network for the three-dimensional gray-matter imaging data. MobileViT combines a lightweight CNN feature extractor with the attention mechanism of the ViT, maintaining performance while reducing model complexity [44]. Based on the Transformer architecture, MobileViT uses the self-attention mechanism to capture global and local image features and model long-range dependencies and global contextual information [45]. The input feature map was unfolded into non-overlapping flattened patches for Transformer-based feature modeling to encode relationships among the patches, as represented by Equation (1):

(1) *X_G_* = *Transformer*(*X_U_*(*p*)),1 ≤ *p* ≤ *P*

After global feature modeling, the output was folded back while maintaining the spatial order of the patches and pixels within each patch. The resulting features were projected into a lower-dimensional channel space through point-wise convolution and concatenated with the input tensor. A convolution operation was then used to fuse the concatenated features and obtain the output of the MobileViT block. To examine the influence of different MobileViT model configurations on classification performance, three configurations within the MobileViT framework were evaluated. Their architectural parameters, including the output channels, MV2 expansion ratios, and Transformer channels, are summarized in Table 4. MobileViT-XS was selected as the feature extraction backbone in the proposed multimodal classification network. The non-imaging features included demographic information, clinical examination scores, and 10 global brain network properties derived from rs-fMRI. These features were first combined into a one-dimensional vector for each participant and then fed into the MLP. MLP is suitable for processing relatively small-scale datasets, and its fully connected layers contain learnable weights [46,47]. In the proposed network, the first linear layer maps the 15 input features to 32 output features. A ReLU activation layer was then applied to introduce nonlinear transformation, as follows:

(2) *y* = max(0,*x*)

The second linear layer maps the 32-dimensional output to 64 features, followed by a ReLU activation. The third linear layer maps the 64 features to 128 dimensions, followed by another ReLU activation. Finally, the fourth linear layer maps the 128 features to 64 dimensions, followed by a final ReLU activation. The resulting 64-dimensional feature representation was used for subsequent multimodal fusion.

Before multimodal model training, the sMRI branch was pretrained using the sMRI-only Group B data. Five-fold cross-validation was applied to the pretraining procedure. The resulting MobileViT-XS sMRI weights were then used to initialize the sMRI branch of the multimodal model. The complete multimodal model was subsequently trained and evaluated on Group A using matched sMRI data together with rs-fMRI-derived global brain network properties, demographic information, and clinical neuropsychological examination scores. The framework adopted feature-level fusion, with the sMRI gray-matter images and non-imaging feature vectors processed by MobileViT and MLP, respectively. Different modalities provide complementary information, and their integration can provide more representative feature information for classification [48].

As shown in Figure 2, the matched sMRI gray-matter images, demographic information, clinical examination scores, and 10 global brain network properties derived from rs-fMRI were used as multimodal inputs for each participant. After feature extraction by the two networks, the feature maps before the final output layer were flattened into one-dimensional vectors. The flattened features from MobileViT and MLP were then concatenated for feature-level fusion [49]. The fused one-dimensional vector was further processed by a fully connected layer, reducing the feature dimension to 256. A ReLU activation function was then applied. Finally, the 256-dimensional feature representation was mapped to a three-dimensional output space to achieve three-class classification of AD, MCI, and NC.

### 2.7. Experimental Settings and Model Evaluation

The experiments were conducted on a workstation equipped with an Intel Core i9-10900X CPU (Intel Corporation, Santa Clara, CA, USA) at 3.70 GHz and an NVIDIA GeForce RTX 3090 GPU (NVIDIA Corporation, Santa Clara, CA, USA). The experimental code was implemented using Python 3.10, CUDA 13.0, and the PyTorch 2.11.0 framework. The network model was trained using the Adam optimizer. The batch size was set to 16, and the learning rate was set to 0.0001 to control the step size of each parameter update and help reduce overfitting. Five-fold cross-validation was used throughout the ADNI model development process, including the sMRI-only pretraining stage and the subsequent multimodal training and evaluation stage. The sMRI-only Group B data were used for pretraining the MobileViT-XS sMRI branch, and the resulting pretrained weights were used to initialize the multimodal model evaluated on Group A. For the independently collected dataset, an 8:2 participant-level split was adopted because of the limited sample size. The pretrained sMRI branch was used for model initialization, and the 80% model-fitting subset was used for dataset-specific fine-tuning. The remaining 20% of participants were held out throughout the fine-tuning process and were used only for final testing. No information from the held-out testing subset was used for model optimization or parameter selection. The data were randomly partitioned at the participant level while considering the diagnostic categories to maintain a relatively consistent class distribution. During pretraining, the number of training epochs for each modality was set to 100. After multimodal fusion, the number of training epochs was set to 50.

To evaluate the classification performance of the experimental model, accuracy, sensitivity, precision, and F1 score were used as performance evaluation metrics [46]. Accuracy refers to the proportion of correctly predicted samples among all samples. In this study, it represents the ratio between the predicted results and the actual labels of the three participant groups. It is calculated as follows:

(3) $$\mathrm{Accuracy}=\frac{\mathrm{TP}+\mathrm{TN}}{\mathrm{TP}+\mathrm{TN}+\mathrm{FP}+\mathrm{FN}}$$

where true positives (TP) represent the number of samples correctly predicted as the positive class; true negatives (TN) represent the number of samples correctly predicted as the negative class; false positives (FP) represent the number of negative samples incorrectly predicted as the positive class; and false negatives (FN) represent the number of positive samples incorrectly predicted as the negative class. The value of accuracy ranges from 0 to 1. A value closer to 1 indicates higher classification accuracy of the model. For the three-class classification task, sensitivity, precision, and F1 score were first calculated separately for each diagnostic category and subsequently summarized using macro averaging, thereby assigning equal weight to AD, MCI, and NC. Sensitivity is commonly used when greater attention is given to important classes. It is calculated as follows:

(4) $$\mathrm{Sensitivity}=\frac{\mathrm{TP}}{\mathrm{TP}+\mathrm{FN}}$$

Precision is an evaluation metric used to assess classification performance. It measures the proportion of true positive samples among all samples predicted as positive. In other words, precision reflects the extent to which negative samples are incorrectly predicted as positive. It is calculated as follows:

(5) $$\mathrm{Precision}=\frac{\mathrm{TP}}{\mathrm{TP}+\mathrm{FP}}$$

The F1 score is a comprehensive metric for evaluating classification performance. It considers both precision and recall. The F1 score is the harmonic mean of precision and recall and is calculated as follows:

(6) F1 Score=2×Precision×RecallPrecision+Recall

In addition to the model performance metrics, statistical hypothesis testing was performed to assess differences in demographic and clinical characteristics among the diagnostic groups. The corresponding *p*-values were reported for variables including age, sex, weight, MMSE score, and CDR score. To further assess whether the observed improvement in classification performance was statistically significant, a paired *t*-test was performed to compare the accuracy of the sMRI-only MobileViT-XS model with that of the multimodal MobileViT-XS + MLP model using the same five-fold cross-validation partitions. The mean difference in accuracy and its corresponding 95% confidence interval were calculated. A two-sided *p*-value of less than 0.05 was considered statistically significant.

## 3. Results

### 3.1. Demographic and Clinical Neuropsychological Measures

The demographic and clinical neuropsychological characteristics of the ADNI dataset and the independently collected dataset are summarized in Table 5. For the ADNI dataset, the participant counts in Table 5 were derived by combining the unique participants from the sMRI-only pre-training pool (Group B) and the multimodal evaluation group (Group A), which were independently partitioned without participant-level overlap. Group B included 776 participants, comprising 147 AD, 325 MCI, and 304 NC participants, whereas Group A included 294 participants, comprising 88 AD, 115 MCI, and 91 NC participants, as shown in Table 1. Accordingly, the combined ADNI dataset included 235 AD, 440 MCI, and 395 NC participants, yielding a total of 1070 unique participants. These values represent the participants retained for the corresponding pre-training and multimodal evaluation analyses and do not represent duplicated counts across the two groups.

The results of the statistical comparisons among the three diagnostic groups are also presented in Table 5. In the ADNI dataset, significant differences were observed among the AD, MCI, and NC groups in age, sex, weight, MMSE score, and CDR score, with *p*-values of <0.001, 0.046, 0.001, <0.001, and <0.001, respectively. In the independently collected dataset, no significant differences were observed in age or sex, with *p*-values of 0.374 and 0.510, respectively. A significant difference was observed in weight, with a *p*-value of 0.013. Significant differences were also observed in MMSE and CDR scores, with both *p*-values < 0.001.

### 3.2. Multimodal Data Classification Results

The classification results obtained using different input modalities and network configurations are presented in Table 6. For single-modality evaluation on sMRI gray matter images, MobileViT-XS achieved the highest performance among the vision backbones, yielding an accuracy of 0.904 ± 0.028, sensitivity of 0.872 ± 0.032, precision of 0.919 ± 0.038, and F1 score of 0.890 ± 0.033. MobileViT-S and MobileViT-XXS followed, with accuracies of 0.882 ± 0.021 and 0.875 ± 0.025, respectively. The baseline MLP model based on non-imaging feature vectors yielded lower performance, reaching an accuracy of 0.812 ± 0.040, sensitivity of 0.797 ± 0.059, precision of 0.779 ± 0.058, and F1 score of 0.786 ± 0.057.

**Figure 3:**
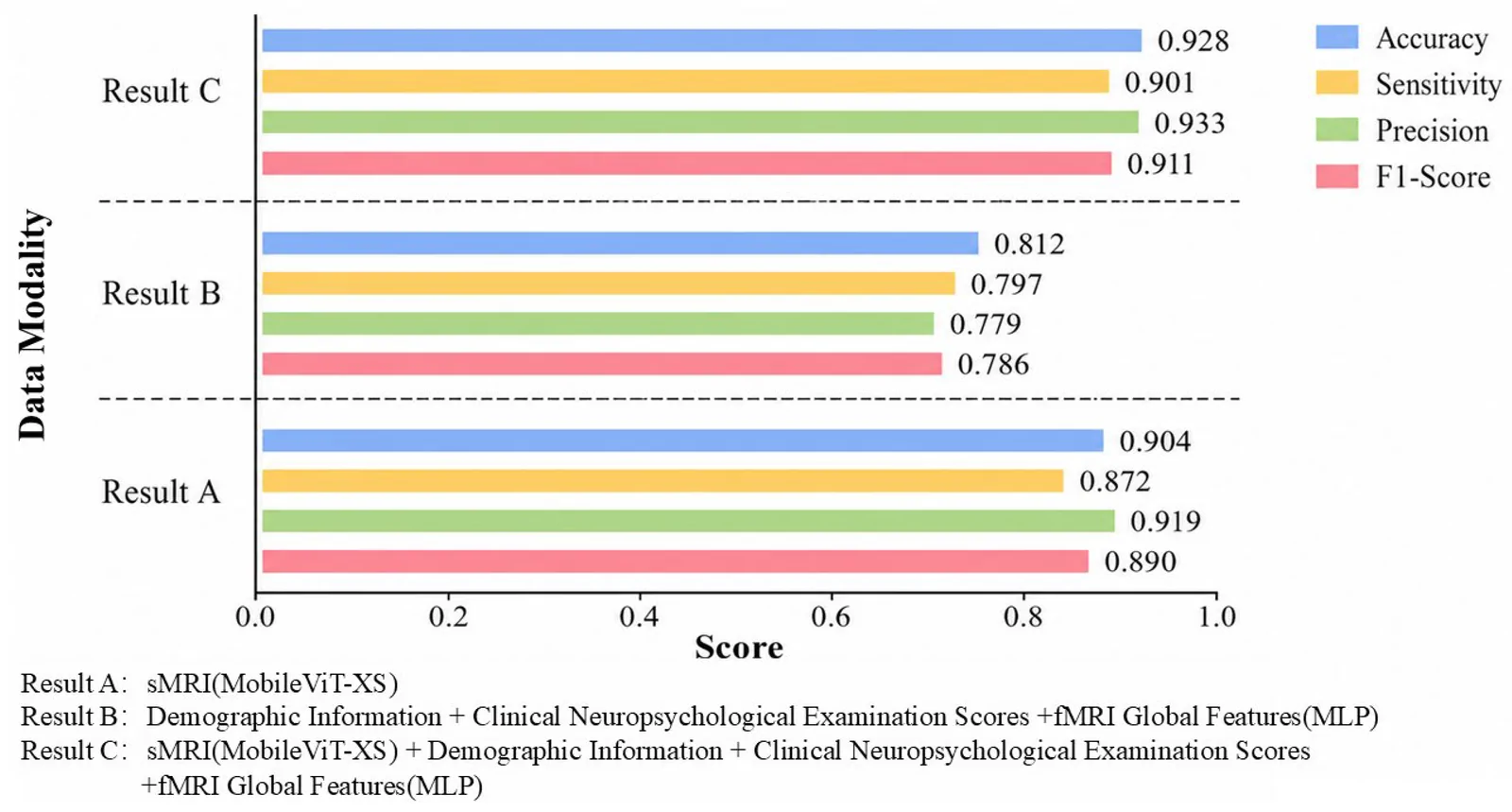
Classification results of different modality data combinations using sMRI gray-matter images under the MobileViT-XS architecture.

**Figure 4:**
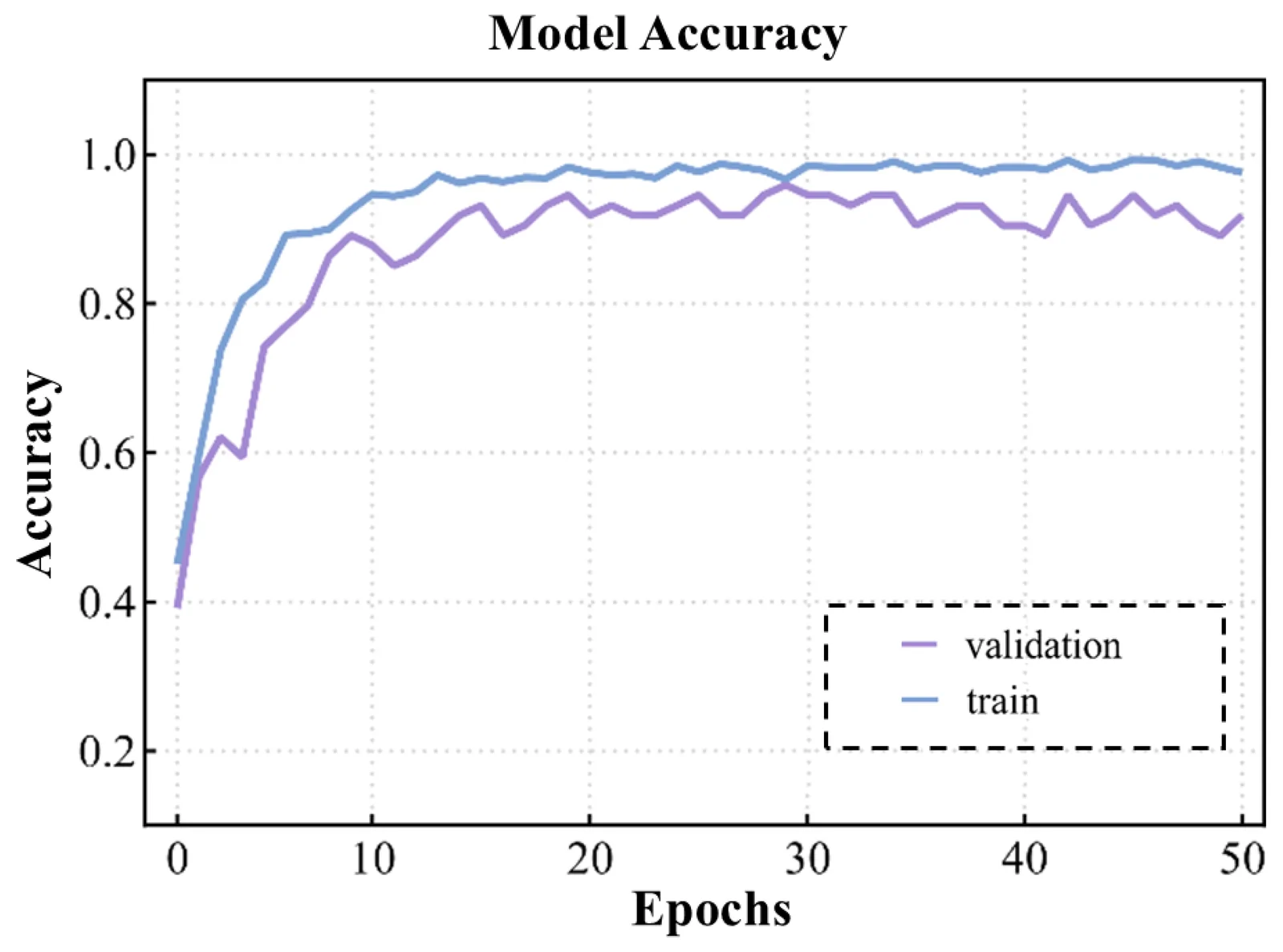
Changes in training and validation accuracy of the multimodal classification network model under the MobileViT-XS and MLP combined architecture.

Following multimodal fusion, the MobileViT-XS + MLP configuration achieved the highest overall accuracy of 0.928 ± 0.010 and precision of 0.933 ± 0.014. In terms of mean sensitivity, MobileViT-XS + MLP reached 0.901 ± 0.014, matching the mean value of MobileViT-S + MLP at 0.901 ± 0.045, but demonstrated a noticeably smaller standard deviation that reflects greater consistency across validation folds. Similarly, although MobileViT-S + MLP achieved a slightly higher mean F1 score of 0.913 ± 0.039 compared to 0.911 ± 0.012 for MobileViT-XS + MLP, MobileViT-XS + MLP consistently exhibited lower standard deviations across all evaluated metrics. As shown in Figure 3, the classification performance varied across different modality combinations. Compared with the sMRI-only MobileViT-XS model, the multimodal MobileViT-XS + MLP model showed a mean fold-wise accuracy improvement of 0.025. Based on a paired *t*-test, this improvement was statistically significant, with a 95% confidence interval of 0.002 to 0.047 and a *p*-value of 0.0391. Overall, MobileViT-XS + MLP provided the most consistent performance across the evaluated metrics owing to its superior a 3ccuracy, highest precision, and consistently higher cross-fold stability, and was therefore selected as the final model configuration. The corresponding training and validation accuracy curves for this configuration are illustrated in Figure 4.

### 3.3. Classification Results of Related AD Diagnostic Methods

The classification performance of the proposed method and recently reported three-class classification of Alzheimer’s disease-related diagnostic groups using multimodal ADNI data is compared in Table 7 [47,48,49,50,51,52,53,54,55,56]. Although the studies differed in their choice of multimodal inputs, sMRI was commonly used as the main imaging modality. Most of the compared methods employed 3D CNNs with attention mechanisms or Transformer-based architectures, making them broadly comparable with the proposed framework. For the ADNI dataset, the proposed method achieved an accuracy of 0.928, a sensitivity of 0.901, a precision of 0.933, and an F1 score of 0.911. These results indicate that the proposed multimodal framework provides competitive performance for three-class classification of AD, MCI, and NC.

**Figure 5:**
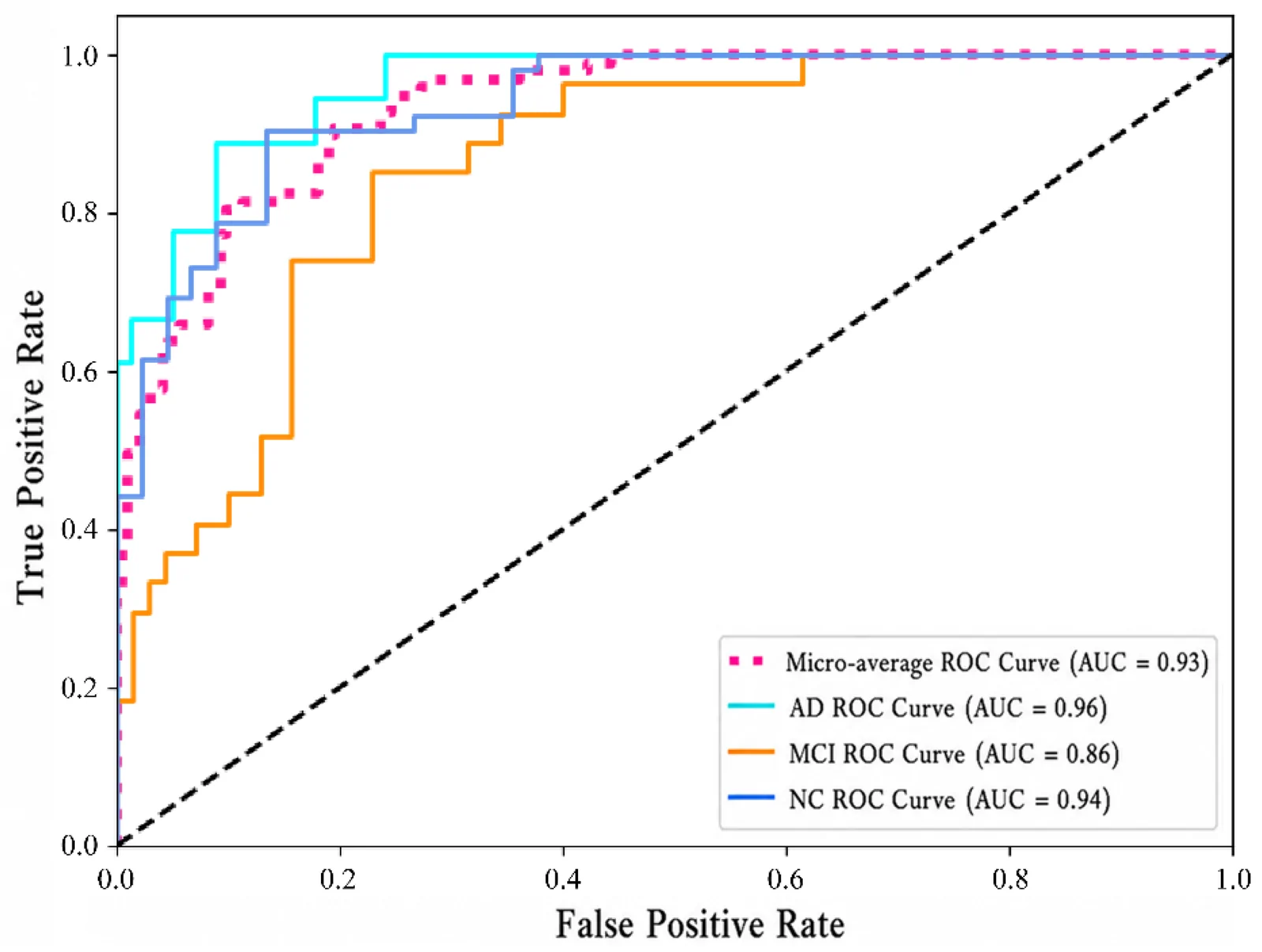
ROC curves and AUC values of the multimodal classification network under the MobileViT-XS architecture.

The ROC curves of the MobileViT-XS-based multimodal model on the ADNI dataset are shown in Figure 5. The AUC values were 0.96 for AD, 0.86 for MCI, and 0.96 for NC, with a macro-averaged AUC of 0.93. The AUC values were calculated using a one-versus-one macro-averaging strategy.

**Figure 6:**
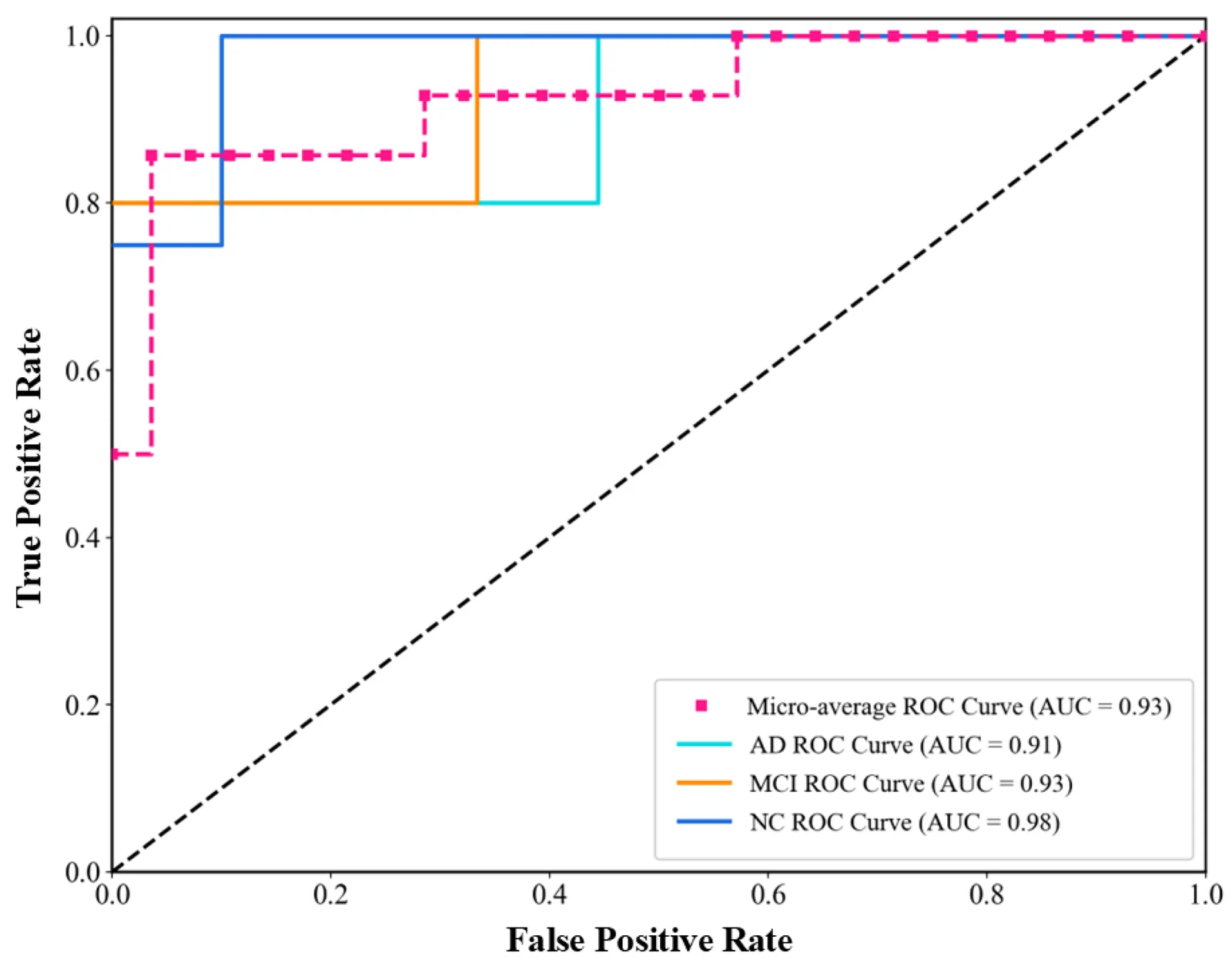
ROC curves and AUC values of the proposed multimodal classification model on the held-out testing subset after dataset-specific fine-tuning.

The model was further evaluated on the held-out testing subset of the independently collected dataset after dataset-specific fine-tuning. The proposed model achieved an accuracy of 0.857, a sensitivity of 0.867, a precision of 0.867, and an F1 score of 0.859 on this held-out testing subset. The corresponding ROC curves are shown in Figure 6. The AUC values were 0.91 for AD, 0.93 for MCI, and 0.98 for NC, with a micro-averaged AUC of 0.93.

### 3.4. Ablation Study

**Figure 7:**
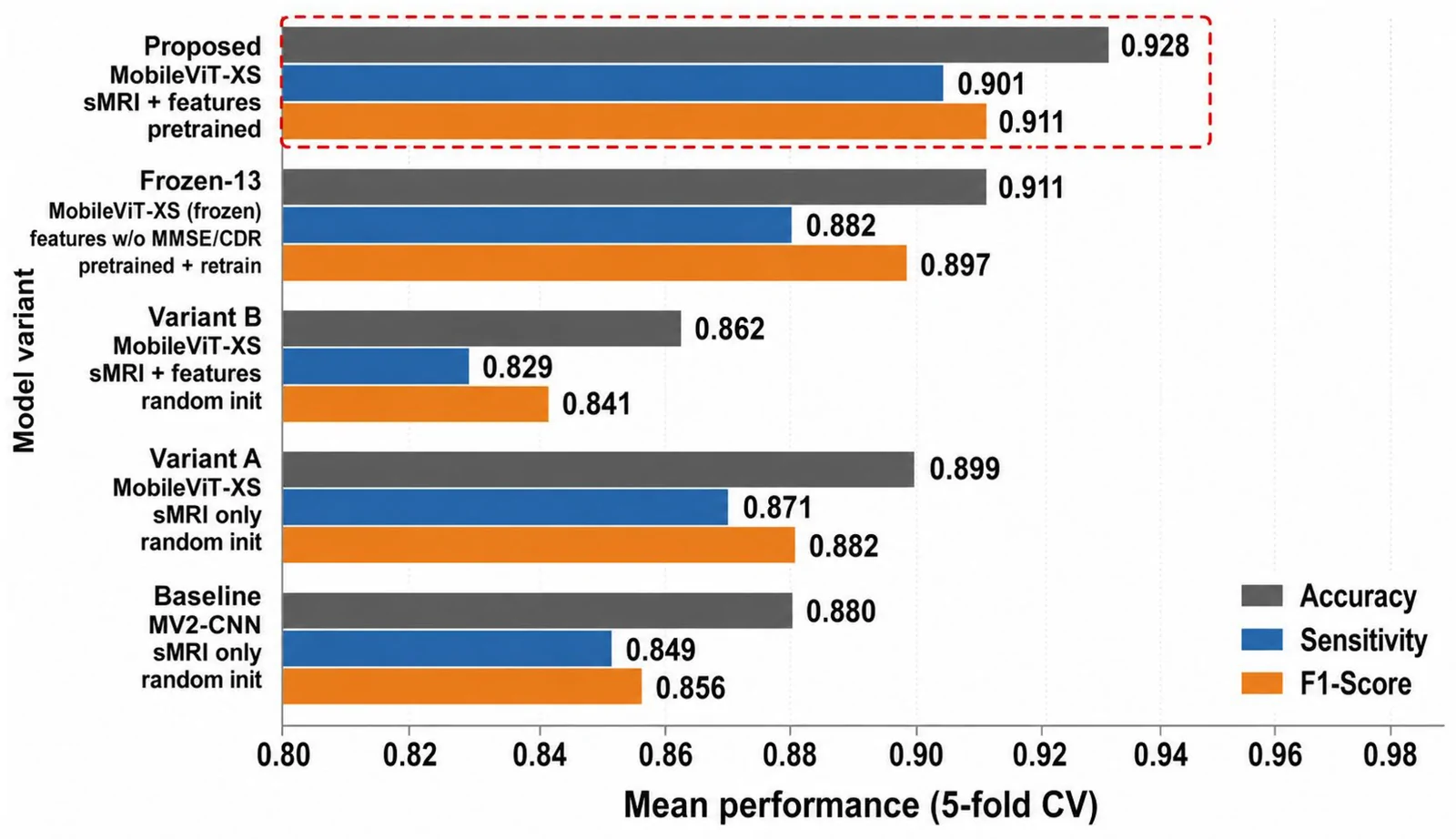
Ablation study results for different model configurations. Accuracy, sensitivity, and F1 score are shown for each model variant. The dashed frame highlights the relevant model configuration(s) for comparison.

**Figure 8:**
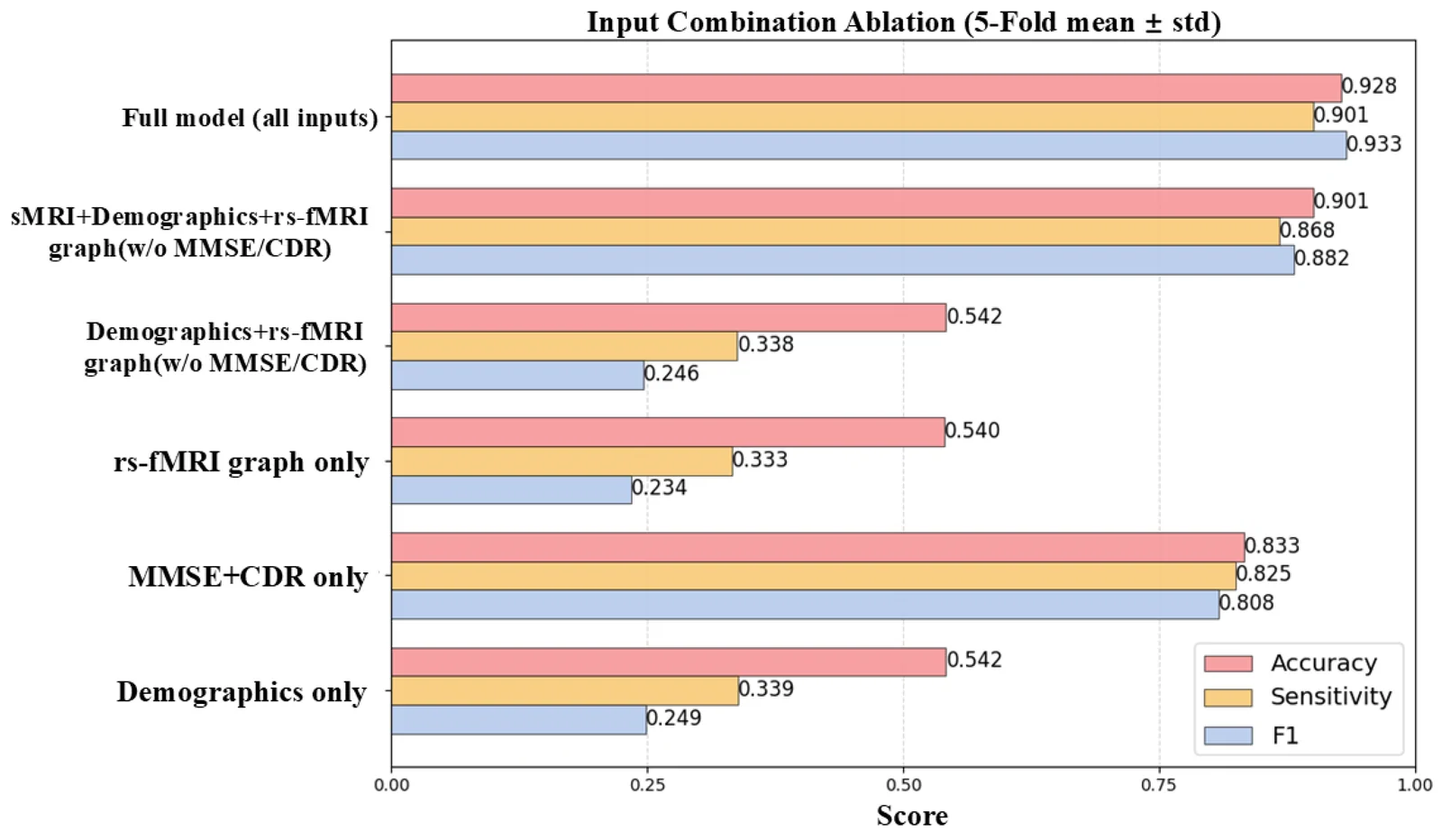
Classification performance under different input feature combinations. Accuracy, sensitivity, and F1 score are shown for each feature configuration.

The ablation results are presented in Figure 7. Replacing the MV2 CNN baseline with MobileViT-XS increased the accuracy from 0.880 to 0.899 and the F1 score from 0.856 to 0.882. The effects of multimodal feature integration and the pretraining strategy are also shown in Figure 7. The fully pretrained multimodal framework achieved an accuracy of 0.928, a sensitivity of 0.901, and an F1 score of 0.911. The input feature ablation results are presented in Figure 8. The model using clinical examination scores alone achieved an accuracy of 0.833. After MMSE and CDR were excluded, the model achieved an accuracy of 0.901. The complete multimodal model achieved an accuracy of 0.928.

### 3.5. Network Parameters and Lightweight Analysis

The proposed multimodal fusion network contained 12.31 million parameters and required approximately 65 GMACs for inference. The sMRI and fMRI feature extraction branches contained approximately 1.95 million and 3.97 million parameters, respectively, while the clinical feature branch contained approximately 0.02 million parameters. The final fusion fully connected layer accounted for 51.8% of the total parameters. As shown in Table 8, the proposed model contained fewer parameters than ResNet-50, 3D ResNet-18, EfficientNet-B4, ViT-Base/16, and Swin-Base.

### 3.6. Model Interpretability and Visual Explanations

**Figure 9:**
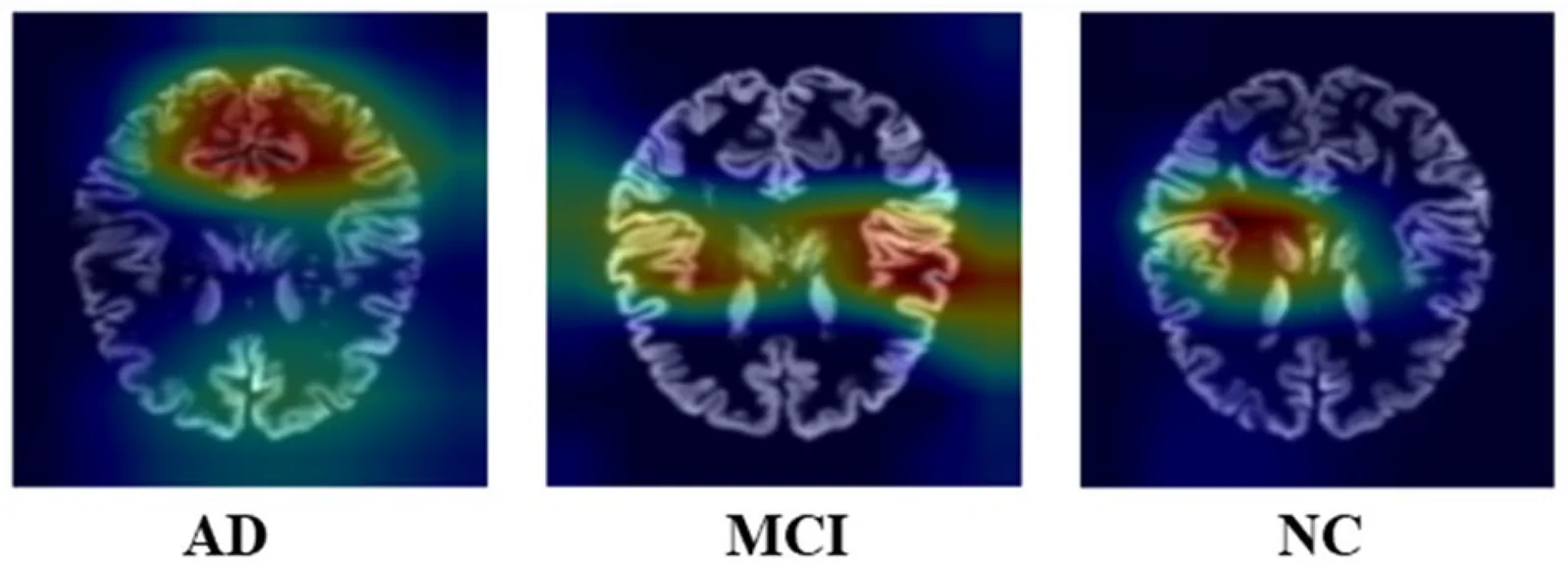
Grad-CAM activation maps of representative AD, MCI, and NC samples. Warmer colors (red and yellow) indicate higher activation intensity, whereas cooler colors (green and blue) indicate lower activation intensity.

To demonstrate the interpretability of the proposed multimodal fusion model and verify its focus on distinct anatomical features, the Gradient-weighted Class Activation Mapping technique, commonly referred to as Grad-CAM, was used to visualize the regions that contributed to the classification results of the proposed multimodal fusion model. As shown in Figure 9, different activation patterns were observed across the three diagnostic groups. For the AD group, the activation regions were relatively concentrated in specific anterior cortical areas. For the MCI group, the activation regions were more dispersed across the hemisphere. For the NC group, the activation regions were mainly localized to different areas of the brain tissue. These results provide a visual representation of the different spatial feature patterns learned by the model for the three diagnostic groups.

**Figure 10:**
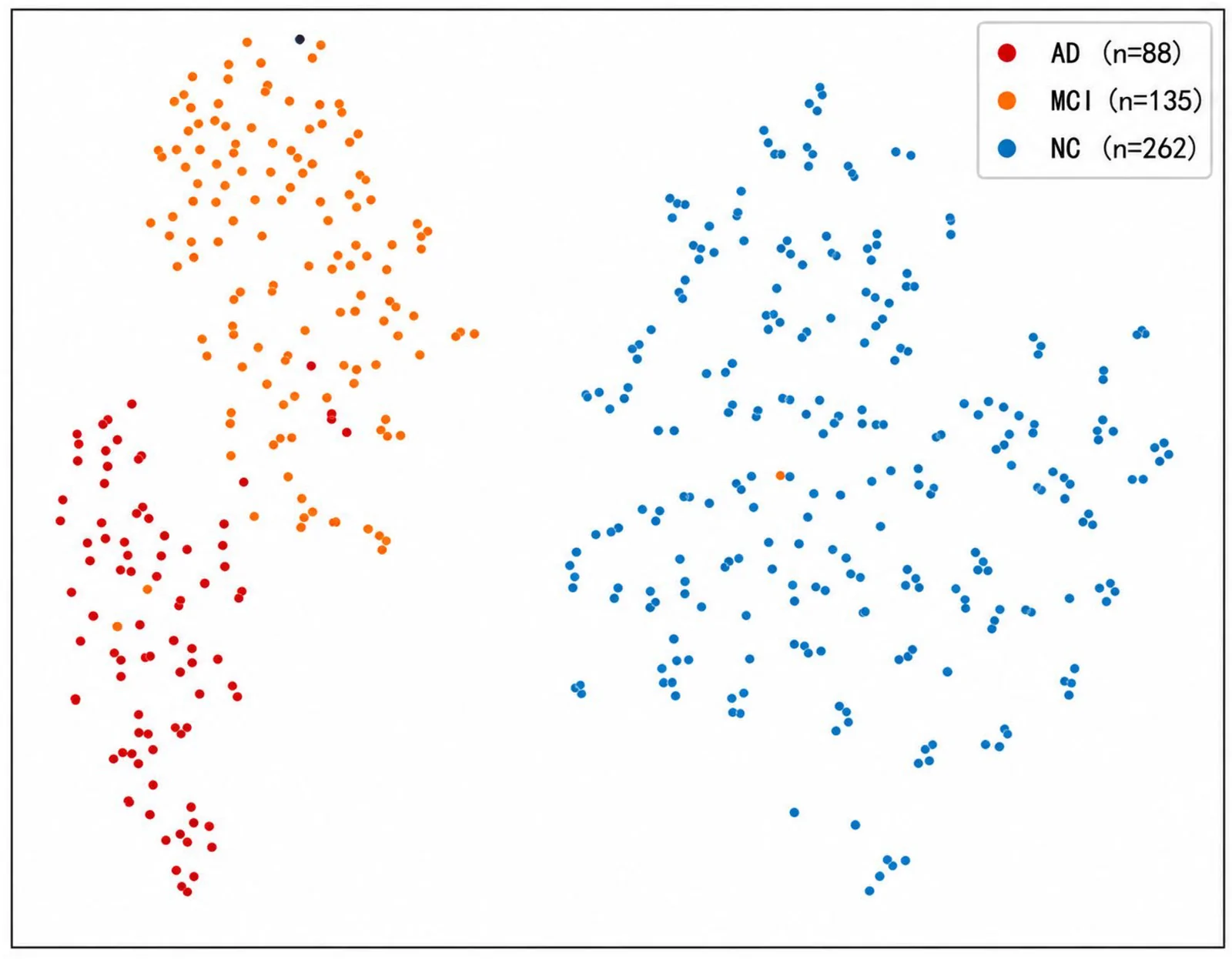
t-SNE visualization of the latent features extracted before classification.

Furthermore, to evaluate the effectiveness of our feature extraction and multimodal fusion strategy, we applied the t-Distributed Stochastic Neighbor Embedding technique, commonly referred to as t-SNE, to the high-dimensional feature vectors extracted from the final fully connected layer prior to classification. As shown in Figure 10, the extracted features formed three distinguishable clusters corresponding to the AD, MCI, and NC groups. The separation between the clusters was particularly evident between the AD and NC groups. These results show that the extracted feature representations contained distinguishable information among the three diagnostic groups.

**Figure 11:**
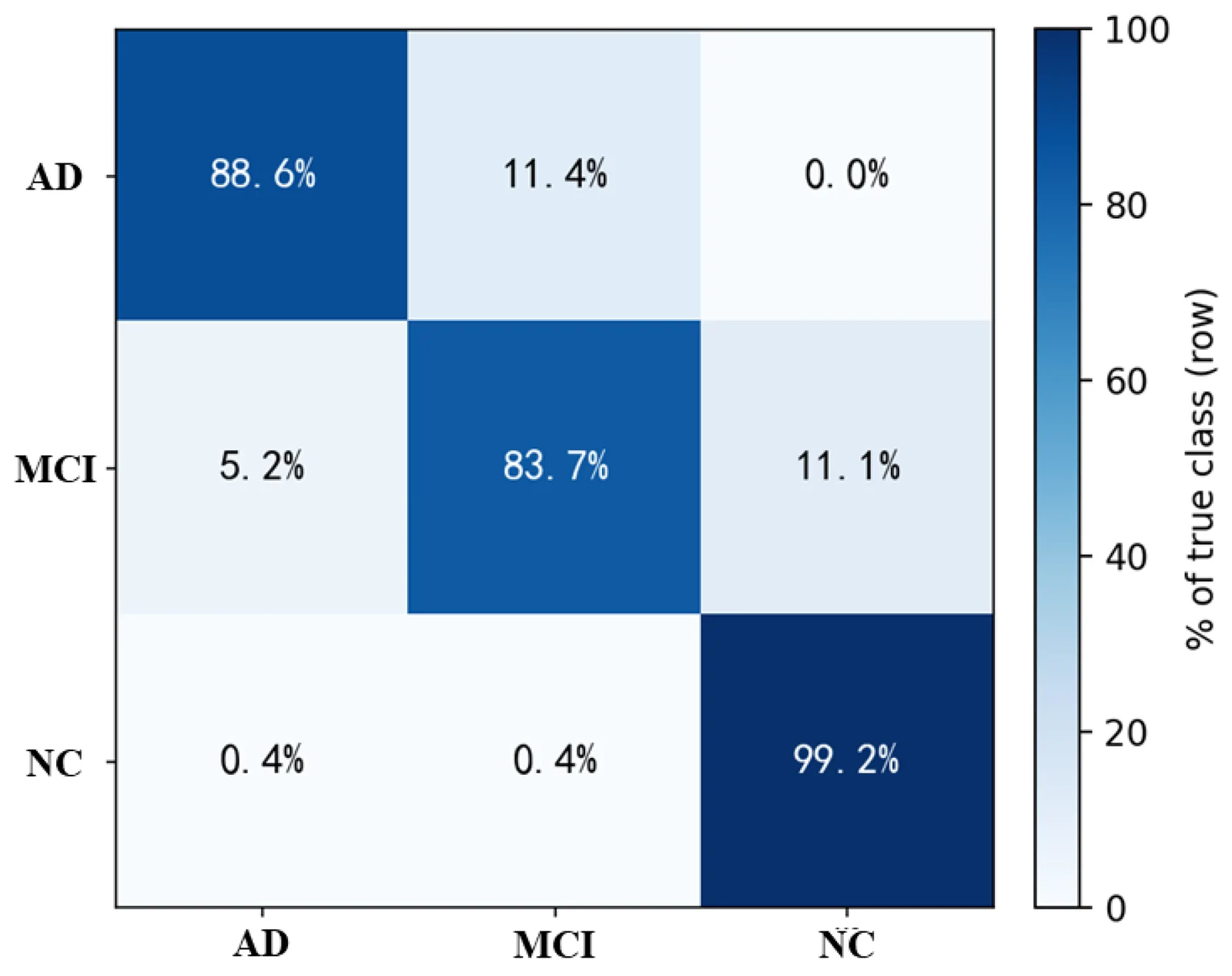
Confusion matrix of the proposed multimodal classification model on the ADNI dataset. The values represent the percentage of predictions within each true diagnostic class.

**Figure 12:**
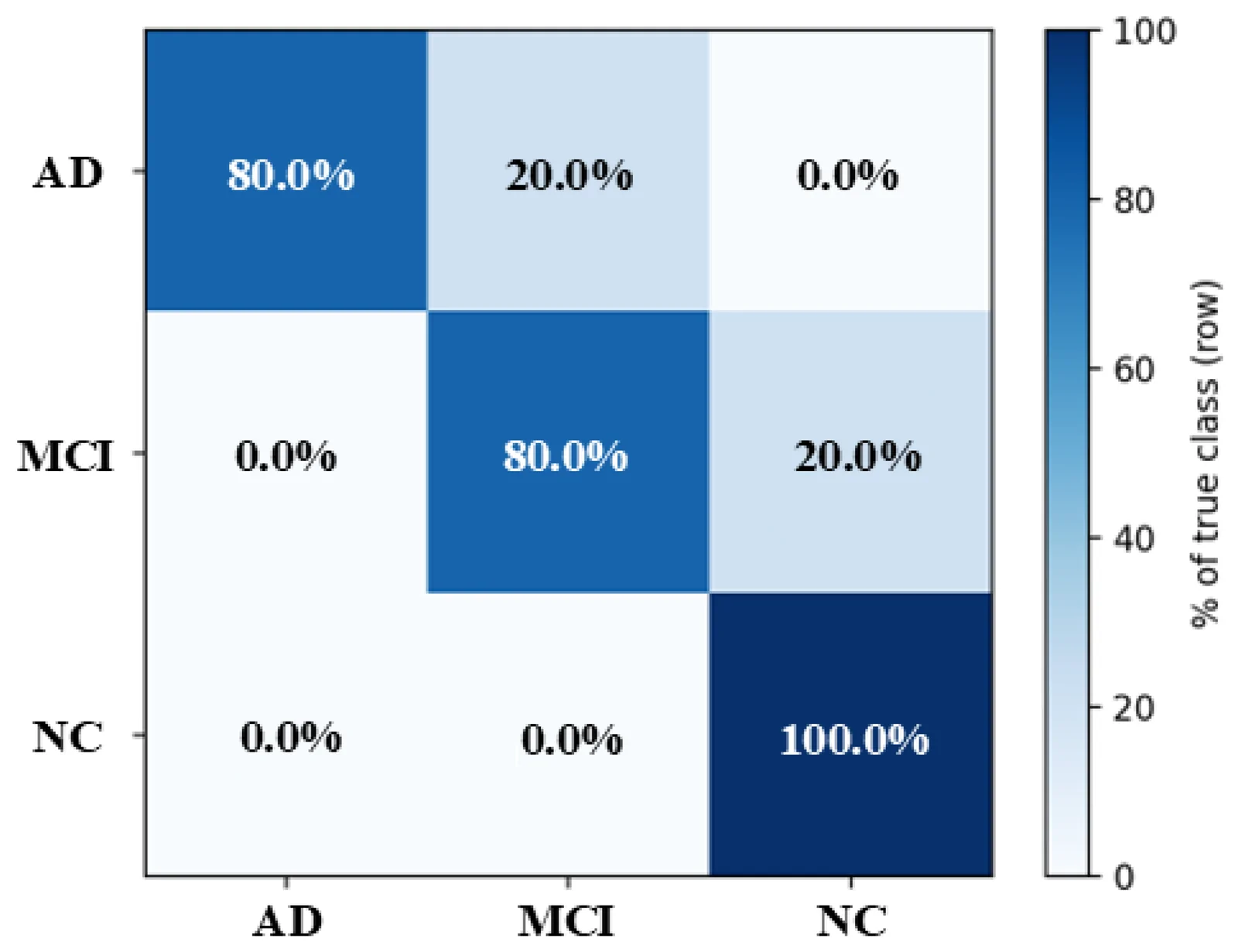
Confusion matrix of the proposed multimodal classification model on the held-out testing subset of the independently collected dataset. The values represent the percentage of predictions within each true diagnostic class.

To further examine the class-wise diagnostic performance of the proposed MobileViT-XS and MLP fusion architecture, the confusion matrices for the ADNI dataset and the independently collected dataset are presented in Figure 11 and Figure 12, respectively. For the ADNI dataset, the NC group showed a true positive rate of 99.2%, while the corresponding value for the AD group was 88.6%. Misclassifications were mainly observed between adjacent diagnostic groups, with 11.4% of AD cases classified as MCI and 11.1% of MCI cases classified as NC.

For the independently collected dataset, the held-out testing subset contained 14 participants, including 5 AD participants, 5 MCI participants, and 4 NC participants. As shown in Figure 12 and Figure 4 of 5 AD participants were correctly classified as AD, whereas 1 was classified as MCI. Similarly, 4 of 5 MCI participants were correctly classified as MCI, whereas 1 was classified as NC. All 4 NC participants were correctly classified. Thus, the corresponding class-specific recalls were 80.0% for AD, 80.0% for MCI, and 100.0% for NC. The corresponding exact 95% confidence intervals were 28.4–99.5% for AD, 28.4–99.5% for MCI, and 39.8–100% for NC.

## 4. Discussion

### 4.1. Classification Performance Analysis of Multimodal Data

To examine the contribution of multimodal fusion, the classification performance obtained from different modality configurations was compared. As shown in Table 6, the overall classification performance of the one-dimensional vector composed of demographic information, clinical examination scores, and 10 global brain network properties derived from rs-fMRI in the MLP network was lower than that of the gray-matter images extracted from sMRI images.

Among the evaluated MobileViT configurations, MobileViT-XS achieved the highest accuracy and precision, while MobileViT-S achieved a slightly higher F1 score. The two configurations showed the same mean sensitivity. The differences among the MobileViT architectures with different network scales mainly lie in model depth and width, number of parameters, and computational complexity [36]. Compared with MobileViT-S, MobileViT-XS uses a smaller network configuration while maintaining higher classification performance in the present experiments. Based on the classification results, MobileViT-XS provided a favorable configuration for classifying the sMRI gray-matter images in the present experiments. The larger number of parameters in MobileViT-S increases model complexity and computational requirements, which may affect training efficiency and model generalization. MobileViT-XXS is the smallest model in the MobileViT family. Based on MobileViT-XS, it further reduces model depth and width, as well as the number of parameters and computational complexity. However, for the three-dimensional sMRI gray-matter images used in this experiment, the images themselves are large and contain complex brain structures, tissue features, and texture information, showing a certain degree of complexity. Although MobileViT-XXS can provide good performance and faster inference speed, it weakens the network’s ability to extract image features. Therefore, MobileViT-XS was selected as the classification model for sMRI gray-matter images because it achieved higher accuracy and precision, while exhibiting smaller standard deviations across the evaluated metrics and maintaining a relatively moderate model scale.

The results showed that multimodal fusion improved classification performance compared with the sMRI-only model. The accuracy increased from 0.904 ± 0.028 for the sMRI-only MobileViT-XS model to 0.928 ± 0.010 for the multimodal MobileViT-XS + MLP configuration. The paired *t*-test further supported this improvement, with a mean accuracy difference of 0.025, a 95% confidence interval of 0.002 to 0.047, and a *p*-value of 0.0391. These results provide statistical evidence of an improvement in classification accuracy under the evaluated five-fold cross-validation setting. The multimodal framework integrates complementary information from sMRI, rs-fMRI-derived features, demographic information, and clinical neuropsychological examination scores, thereby providing a more comprehensive representation for three-class classification.

### 4.2. Comparison of Classification Performance of AD-Related Methods

Compared with previously reported methods using multimodal ADNI data, the proposed framework achieved competitive classification performance. The accuracy obtained in the present study was 0.928, which was higher than the values reported by several compared studies, including 0.790 reported by Venugopalan et al. and 0.838 reported by Wang et al. The overall AUC of 0.93 was also slightly higher than the AUC of 0.924 reported by Wang et al. These comparisons place the performance of the proposed framework within the higher range of the reported results. As shown in Table 7, the study by Venugopalan J et al. reported an accuracy of 0.79 using a multimodal framework combining sMRI and electronic health records [49]. Wang J X et al. reported an accuracy of 0.8378 and an AUC of 0.924 using a 3D CNN and Transformer-based framework integrating sMRI and gene sequencing data [50].

Multimodal graph neural networks and advanced Transformer architectures have been widely applied in the early diagnosis of AD. For example, Sun et al. reported an accuracy of 0.737 using a meta-learning-based graph convolutional network [48]. Jann et al. reported an accuracy of 0.84 using 3D CNN-based analysis of rs-fMRI and Tau PET data [51]. Wang et al. reported an accuracy of 0.842 using a Transformer-based framework for fMRI analysis [53]. Bahroun et al. reported an accuracy of 0.923 using a hybrid framework combining RNN and ViT branches [55], which was close to the accuracy obtained in the present study. Fu et al. reported accuracies of 0.8772 using graph convolutional network-based analysis of functional connectomes [54].

The studies by Chen H et al. and Zhang J et al. both used attention mechanisms as the main research method in their models. Chen H et al. proposed an attention-based multimodal fusion network [52]. In their method, sMRI images and positron emission tomography (PET) images were converted into feature vectors with the same dimensionality, while demographic information and clinical data were also transformed into feature vector representations. Channel attention and spatial attention mechanisms were then used to fuse the two types of feature vectors for three-class classification, achieving an accuracy of 84.1%. Zhang J et al. also used an attention mechanism [47]. They processed image data, including sMRI images and fluorodeoxyglucose positron emission tomography (FDG-PET) images, using cascaded dilated convolution. Non-imaging data, including cerebrospinal fluid (CSF) information, were learned through an encoder. Image and non-image information were then integrated using a cross-modal attention method for classification [57]. Both studies adopted feature-level fusion, which was consistent with the fusion strategy used in this experiment for multimodal data. However, their reported classification performance was lower than that of the proposed method. Notably, the study by Zheng S et al. reported an accuracy of 0.9231 using a multimodal graph convolutional network [56], which was close to the result obtained in the present study. Overall, the proposed method demonstrated competitive classification performance compared with the methods reported in the above studies.

### 4.3. Ablation Analysis

The ablation analysis provides a controlled evaluation of the main components of the proposed framework. Replacing the MV2 CNN baseline with MobileViT-XS increased the accuracy from 0.880 to 0.899 and the F1 score from 0.856 to 0.882. This improvement indicates that the selected MobileViT architecture provided more effective feature extraction for the sMRI classification task under the evaluated conditions.

Further performance improvements were obtained after the additional feature inputs and the pretraining strategy were incorporated. The complete model achieved an accuracy of 0.928, a sensitivity of 0.901, and an F1 score of 0.911. These results indicate that the improvement was not attributable to the backbone architecture alone, but was also associated with the additional feature information and the pretrained initialization used in the proposed framework.

The input combination analysis further showed that removing MMSE and CDR reduced the accuracy from 0.928 to 0.901. This reduction suggests that the clinical neuropsychological measures contributed useful information to the multimodal representation. Overall, the ablation results provide internal evidence for the contribution of the evaluated architecture, multimodal inputs, and pretraining strategy.

### 4.4. Lightweight Model Analysis

The proposed multimodal network contained 12.31 M parameters and required approximately 65 GMACs for inference, which was lower than the parameter counts reported for the mainstream backbone architectures listed in Table 8. The parameter distribution further indicates that the fusion component represented a substantial proportion of the overall model size.

These results show that the use of MobileViT-XS substantially limited the parameter scale of the feature extraction component within the proposed framework. At the same time, the relatively large proportion of parameters in the final fusion layer indicates that the computational burden was not determined solely by the image feature extraction backbone. Therefore, although the proposed network was relatively lightweight compared with the backbone architectures listed in Table 8, further reduction in the fusion layer could be considered when computational efficiency is a primary concern.

The lightweight architecture is particularly relevant to the design objective of the present study, in which multimodal information was integrated while avoiding the use of a substantially larger backbone network. However, the parameter reduction should be considered together with the classification results rather than as an independent indicator of model effectiveness.

### 4.5. Model Interpretability and Class-Wise Performance

The Grad-CAM visualization showed different spatial activation patterns across the AD, MCI, and NC groups. Higher activation was observed in different brain regions across the three diagnostic groups, providing qualitative information about the spatial features emphasized by the model during classification. These visualizations provide an additional perspective on the learned image representations and help illustrate that different spatial patterns were associated with the model outputs.

The t-SNE visualization showed three distinguishable clusters corresponding to the AD, MCI, and NC groups. This finding is consistent with the classification results and suggests that the learned latent representation contained class-related information. The relatively clear separation between the diagnostic groups also provides visual support for the discriminative characteristics of the extracted multimodal features.

The confusion matrices further showed that classification errors were mainly observed between adjacent diagnostic categories. In the ADNI dataset, errors were particularly observed between AD and MCI and between MCI and NC. A similar pattern was observed in the independently collected dataset. The repeated occurrence of errors involving MCI suggests that classification performance was more challenging for the intermediate diagnostic category than for the NC group.

These visualization and class-wise analyses provide complementary evidence for the behavior of the proposed model. However, the Grad-CAM results should be interpreted as qualitative descriptions of model activation rather than evidence of causal relationships between specific brain regions and disease pathology.

### 4.6. Held-Out Testing After Dataset-Specific Fine-Tuning

A decrease in classification performance was observed when the model was evaluated on the independently collected dataset. The accuracy decreased from 0.928 in the ADNI cross-validation results to 0.857 on the held-out testing subset. Similarly, sensitivity decreased from 0.901 to 0.867, precision decreased from 0.933 to 0.867, and the F1 score decreased from 0.911 to 0.859. This reduction was observed even though the same preprocessing procedures were applied to the two datasets.

The difference in performance may be related to differences between the two data sources. Although the preprocessing procedures were kept consistent and the testing data from both datasets contained matched multimodal information, the two datasets were not identical in scanner characteristics, acquisition protocols, and participant characteristics. Such differences may result in variations in the distributions of the extracted imaging and non-imaging features and may therefore affect the performance of a model trained using one data source when it is applied to another.

Another factor that should be considered is the difference in the training setting between the two datasets. The ADNI evaluation was performed using five-fold cross-validation within the dataset, whereas the independently collected dataset was evaluated using a participant-level 8:2 training-testing split with a held-out testing subset. Because of the limited sample size of the independently collected dataset, the number of participants available for independent testing was relatively small. Consequently, the observed performance reduction may have been affected by both cross-dataset differences and the limited size of the independent testing subset.

The independently collected dataset was divided at the participant level into an 80% model-fitting subset and a 20% held-out testing subset. The pretrained MobileViT-XS sMRI branch obtained from the ADNI sMRI-only pre-training stage was used for model initialization, and the 80% model-fitting subset was used for dataset-specific fine-tuning. The remaining 20% of participants was kept separate from model fitting and parameter selection and was used exclusively for final testing.

On the held-out testing subset, the proposed multimodal model achieved an accuracy of 0.857, a sensitivity of 0.867, a precision of 0.867, and an F1 score of 0.859. These results further demonstrate the classification capability of the proposed framework on independently collected participants after dataset-specific fine-tuning.

Overall, the results support the applicability of the proposed multimodal framework to independently collected data following dataset-specific fine-tuning. Further evaluation using larger and more diverse multicenter datasets can provide additional evidence regarding the robustness of the framework across broader data distributions.

## 5. Conclusions

In conclusion, a multimodal classification framework was developed for the three-class classification of AD, MCI, and NC by combining MobileViT with an MLP-based fusion network. The framework integrated sMRI gray matter features with demographic information, clinical neuropsychological examination scores, and rs-fMRI-derived global brain network properties to exploit complementary information across imaging and non-imaging modalities.

The experimental results showed that multimodal fusion improved classification performance compared with single-modality models, and the MobileViT-XS-based framework achieved the highest performance among the evaluated MobileViT configurations. On the ADNI dataset, the proposed framework achieved an accuracy of 0.928, a sensitivity of 0.901, a precision of 0.933, and an F1 score of 0.911. On the held-out testing subset of the independently collected dataset after dataset-specific fine-tuning, the corresponding values were 0.857, 0.867, 0.867, and 0.859, respectively. These results describe the classification performance obtained on the held-out testing subset after dataset-specific fine-tuning.

The results indicate that the proposed framework achieved competitive classification performance on the ADNI dataset and maintained effective three-class classification performance on the held-out testing subset of the independently collected dataset after dataset-specific fine-tuning. Overall, the framework provides a potential approach for multimodal computer-aided classification of AD, MCI, and NC.

## 6. Limitations and Future Directions

Although the proposed framework achieved competitive classification performance on the ADNI dataset, several limitations should be considered. First, differences between the ADNI dataset and the independently collected dataset may have affected the transferability of the learned representations. Variations in data sources and acquisition settings may introduce distribution differences between the datasets and consequently affect classification performance on independent data. Second, the independently collected dataset contained a limited number of participants, which may have increased the variability of the held-out testing results. In addition, the ADNI dataset was evaluated using five-fold cross-validation, whereas an independent participant-level 8:2 training-testing split was used for the independently collected dataset. Therefore, the difference in evaluation settings should also be considered when interpreting the reduction in performance observed on the independently collected dataset. Further validation using larger and more diverse independently collected datasets is needed to assess the robustness and generalizability of the proposed framework.

Future studies could further evaluate the proposed framework using larger multicenter datasets and more diverse participant populations. Data harmonization across different acquisition settings could also be investigated to reduce potential distribution differences and improve the transferability of the learned representations. In addition, further investigation of multimodal feature interactions may help improve the discrimination of adjacent diagnostic categories, particularly AD, MCI, and NC. Future studies should also address the challenge of monitoring training dynamics in highly parameterized 3D medical imaging pipelines and could further integrate GM, WM, and rs-fMRI features within a unified cross-modal framework, followed by validation of the proposed model on larger, independent multimodal datasets. Overall, the present findings support the potential of combining lightweight sMRI representations with functional and clinical neuropsychological examinations for three-class AD classification, while further validation on independent datasets remains necessary.

## Figures and Tables

**Table 1:** Distribution of participants in the ADNI and independently collected datasets.

Group	Pre-Training Pool (Group B: sMRI Only)	Multimodal Evaluation Cohort (Group A: sMRI + fMRI + Clinical)	Independently Collected Dataset
AD	147 participants (252 scans)	88 participants (88 matched scan sets)	23 participants
MCI	325 participants (480 scans)	115 participants (115 matched scan sets)	26 participants
NC	304 participants (651 scans)	91 participants (91 matched scan sets)	18 participants

**Table 2:** Summary of rs-fMRI scanning parameters for the three participant groups.

Group	TR (ms)	TE (ms)	Slice Thickness (mm)
AD	3000	12	4.0
		30	3.3
	3400	12	3.0
		13	4.0
MCI	2250	30	3.3
	3000	12	4.0
		30	3.3
	3001	30	3.3
	3004	30	3.3
	3005	30	3.3
	3025	30	3.3
	3400	12	4.0
		13	4.0
	4000	13	4.0
NC	3000	12	4.0
		30	3.4
	3001	30	3.3
	3400	12	4.0
		13	4.0

AD, Alzheimer’s disease. MCI, Mild cognitive impairment. NC, normal controls. TR, repetition time. TE, echo time.

**Table 3:** Definitions of global network properties in graph theory.

Global Network Property and Symbol	Definition
Normalized clustering coefficient (γ)	Measures the degree of connection density among nodes in a network. It is defined as the ratio of the clustering coefficient of the observed network to that of a random network.
Normalized characteristic path length (λ)	Describes information transmission between nodes in a network and is used to measure the overall transmission efficiency of the network.
Small-worldness (σ)	Describes a network that is more clustered than a random network but has a characteristic path length similar to that of a random network (σ = γ/λ).
Clustering coefficient (C_p_)	Measures the proportion of actual connections among the nearest neighbors of a node relative to the maximum possible number of connections.
Characteristic path length (L_p_)	Measures the average minimum number of connections required to transmit information between any two nodes in a connected network.
Local efficiency (E_loc_)	Measures the efficiency of information exchange within local clustered regions of the network.
Global efficiency (E_g_)	Measures the efficiency of information exchange across the whole network.
Assortativity	Describes the tendency of nodes with similar degree values to be connected to each other.
Synchronization	Describes the similarity of changes in node degrees when the network is perturbed.
Hierarchy	Measures how sparsely hub nodes are connected, rather than being locally clustered.

**Table 4:** Configuration information of the three MobileViT network architecture scales.

MobileViT Network Architecture		Layer				
		Layer1	Layer2	Layer3	Layer4	Layer5
MobileViT-S	out channels	32	64	96	128	160
	MV2 exp	4	4	4	4	4
	Transformer channels	-	-	144	192	240
MobileViT-XS	out channels	32	48	64	80	96
	MV2 exp	4	4	4	4	4
	Transformer channels	-	-	96	120	144
MobileViT-XXS	out channels	16	24	48	64	80
	MV2 exp	2	2	2	2	2
	Transformer channels	-	-	64	80	96

**Table 5:** Demographic and clinical neuropsychological characteristics of the ADNI and independently collected datasets.

Dataset	ADNI				
	AD	MCI	NC	*F*/χ^2^	*p*
**Number**	235	440	395		
**Age**	75.65 ± 7.73	73.15 ± 7.50	75.59 ± 5.60	16.367 *	<0.001
**Sex (male/female)**	141/94	258/182	203/192	6.149 ^#^	0.046
**Weight**	73.36 ± 14.01	77.95 ± 16.28	76.29 ± 15.64	6.668 *	0.001
**MMSE**	22.55 ± 2.83	27.44 ± 2.07	29.03 ± 1.08	799.197 *	<0.001
**CDR**	0.82 ± 0.36	0.49 ± 0.13	0.01 ± 0.12	872.855 *	<0.001
	**The First Hospital of Jilin University**				
**Number**	23	26	18		
**Age**	66.70 ± 7.04	65.30 ± 6.82	63.44 ± 8.17	1.00 *	0.374
**Sex (male/female)**	9/14	8/18	4/14	1.35 ^#^	0.510
**Weight**	70.10 ± 14.58	65.33 ± 10.30	76.96 ± 9.84	4.61 *	0.013
**MMSE**	17.17 ± 7.65	25.22 ± 2.45	27.94 ± 2.18	27.50 *	<0.001
**CDR**	1.52 ± 0.83	0.56 ± 0.25	0.19 ± 0.25	38.36 *	<0.001

AD, Alzheimer’s disease. MCI, Mild cognitive impairment. NC, normal controls. MMSE, Mini-Mental State Examination; CDR, Clinical Dementia Rating. *, one-way analysis of variance (ANOVA); *p*, *p*-value; ^#^, χ^2^ test.

**Table 6:** Comparison of classification results using different modality data.

Model		Accuracy	Sensitivity	Precision	F1 Score
sMRI	MobileViT-S	0.882 ± 0.021	0.857 ± 0.022	0.882 ± 0.030	0.867 ± 0.025
	MobileViT-XS	**0.904 ± 0.028**	**0.872 ± 0.032**	**0.919 ± 0.038**	**0.890 ± 0.033**
	MobileViT-XXS	0.875 ± 0.025	0.847 ± 0.023	0.877 ± 0.039	0.859 ± 0.031
MLP		0.812 ± 0.040	0.797 ± 0.059	0.779 ± 0.058	0.786 ± 0.057
Multimodal fusion	MobileViT-S + MLP	0.907 ± 0.027	0.901 ± 0.045	0.929 ± 0.024	**0.913 ± 0.039**
	MobileViT-XS + MLP	**0.928 ± 0.010**	**0.901 ± 0.014**	**0.933 ± 0.014**	0.911 ± 0.012
	MobileViT-XXS + MLP	0.866 ± 0.021	0.833 ± 0.027	0.875 ± 0.031	0.850 ± 0.031

Paired *t*-test comparison between the sMRI-only MobileViT-XS model and the multimodal MobileViT-XS + MLP model showed a mean accuracy difference of 0.025 (95% CI: 0.002–0.047, *p* = 0.0391). Bold values indicate the best results for the corresponding evaluation metrics.

**Table 7:** Comparison with literature studies.

Reference	Method	Accuracy	Sensitivity	Precision	F1 Score
Zhang J et al. [47]	Dilated convolution and attention mechanism	0.6403	0.6385	0.8200	0.6185
Sun X et al. [48]	Meta-learning GCN	0.737	-	-	-
Venugopalan J et al. [49]	3D CNN and autoencoder	0.790	0.790	0.790	0.790
Wang J X et al. [50]	3D CNN and Transformer architecture	0.8378	0.7778	0.8750	0.824
Jann K et al. [51]	3D CNN	0.840	-	-	-
Chen H et al. [52]	3D CNN and attention mechanism	0.8410	-	-	-
Wang B C et al. [53]	ViT	0.842	-	-	-
Fu Y et al. [54]	Graph convolutional network (GCN)	0.8772	-	-	0.880
Bahroun S et al. [55]	Hybrid RNN and ViT	0.923	0.864	0.881	0.872
Zheng S et al. [56]	Graph convolutional network (GCN)	0.9231	-	-	-
Ours (ADNI)	MobileViT-XS and MLP-based multimodal fusion	**0.928**	**0.901**	**0.933**	**0.911**
Ours (Held-out testing after dataset-specific fine-tuning)	MobileViT-XS and MLP-based multimodal fusion	0.857	0.867	0.867	0.859

An en dash (–) indicates that data are not available.Bold values indicate the best results for the corresponding evaluation metrics.

**Table 8:** Comparison of parameter scale with mainstream backbones.

Model	Parameters	Typical Application
Ours (Multimodal Fusion)	12.31 M	sMRI + fMRI + Feature Multimodal classification
ResNet-50	25.6 M	2D Image classification benchmark
3D ResNet-18	33.2 M	Video/Medical 3D volumetric data
EfficientNet-B4	19.3 M	Representative lightweight CNN
ViT-Base/16	86.6 M	Standard Transformer
Swin-Base	88 M	Hierarchical Transformer

## Data Availability

The data that support the findings of this study are available from the Alzheimer’s Disease Neuroimaging Initiative (ADNI) database (adni.loni.usc.edu), a publicly available resource. Restrictions apply to the availability of these data, which were used under license for this study. Data are available at adni.loni.usc.edu upon request and with permission of ADNI. The other part of the data used in this study is from the First Hospital of Jilin University. Due to privacy or ethical restrictions, the original neuroimaging data is not publicly available.
